# Supplementary material for: Novel Magnetic Inorganic Composites: Synthesis and Characterization
Source: Polymers (Basel). 2021 Apr 15;13(8):1284. doi: 10.3390/polym13081284 (PMC8071178; doi:10.3390/polym13081284)
Supplement: Supplementary file 1 [file polymers-13-01284-s001.zip › polymers-1170621-supplementary.pdf]

SUPPLEMENTARY MATERIALS

Novel magnetic inorganic composites: synthesis and characterization

*Marco Natali, Sergio Tamburini, Roberta Bertani, Daniele Desideri, Mirto Mozzon, Daniele Pavarin, Federico Spizzo, Lucia Del Bianco, Federico Zorzi, and Paolo Sgarbossa**

List of supplementary figures:

- **Figure S1.** Full-scale XRD profile of the **G1** matrix.

- **Figure S2.** Full-scale XRD profile of the **G2** matrix.

- **Figure S3.** Full-scale XRD profile of the isotropic **$SrFe_{12}O_{19}$** particles.

- **Figure S4.** Full-scale XRD profile of the anisotropic **$SrFe_{12}O_{19}$** particles.

- **Figure S5.** Full-scale XRD profile of the **G1is_11** composite.

- **Figure S6.** Full-scale XRD profile of the **G1isM_11** composite.

- **Figure S7.** Full-scale XRD profile of the **G2anis_6** composite.

- **Figure S8.** Full-scale XRD profile of the **G2anisM_6** composite.

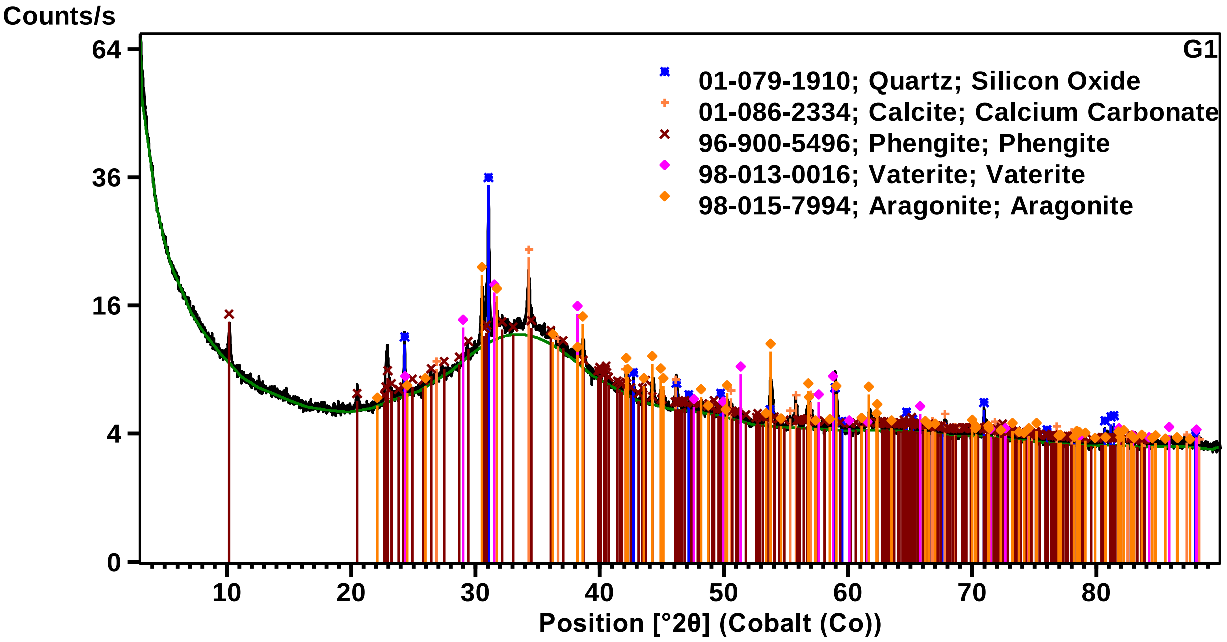

Figure S1. Full-scale XRD profile of the **G1** matrix.

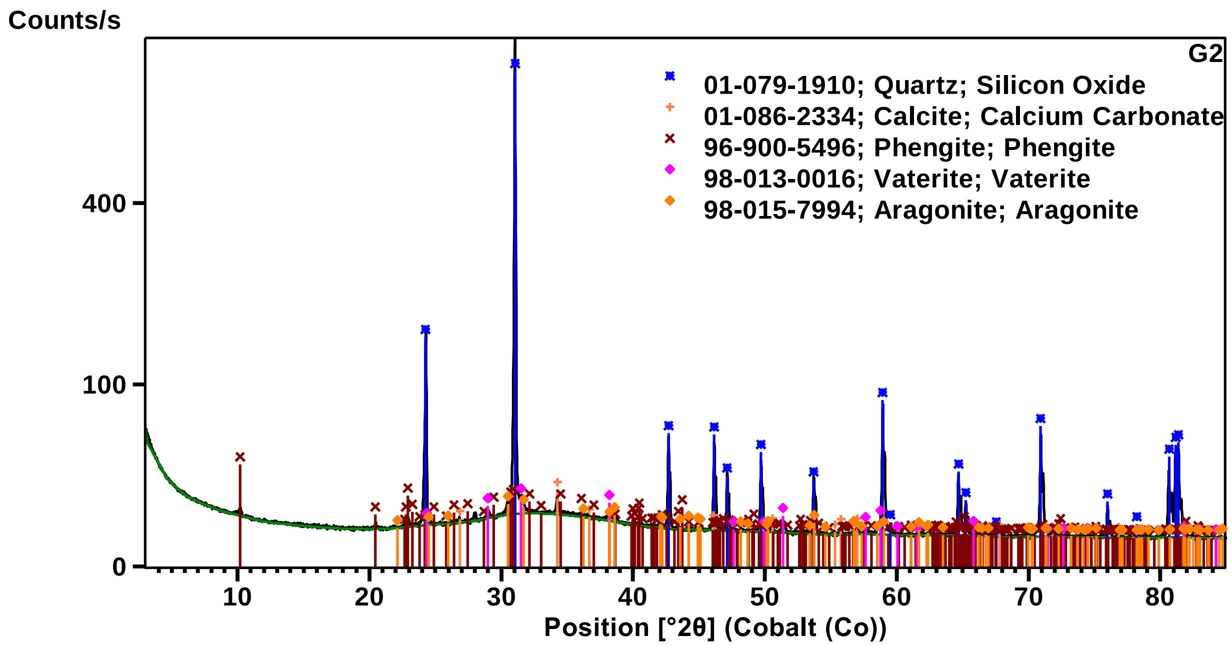

Figure S2. Full-scale XRD profile of the **G2** matrix.

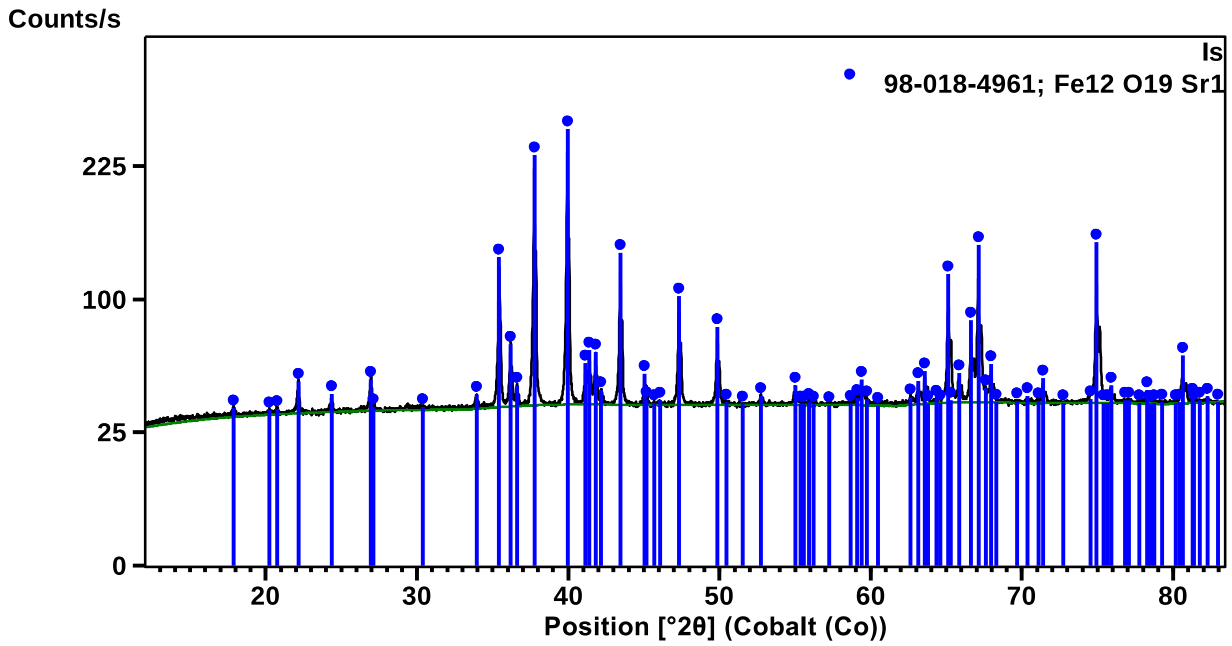

Figure S3. Full-scale XRD profile of the isotropic $\mathbf{SrFe_{12}O_{19}}$ particles.

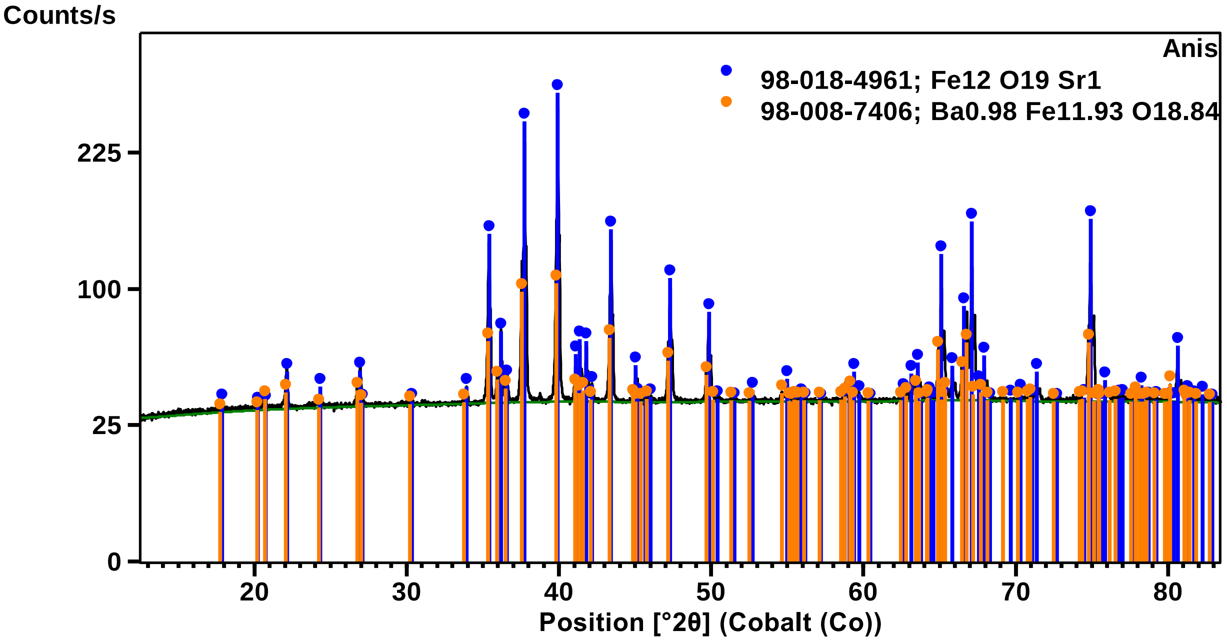

Figure S4. Full-scale XRD profile of the anisotropic $\mathbf{SrFe_{12}O_{19}}$ particles.

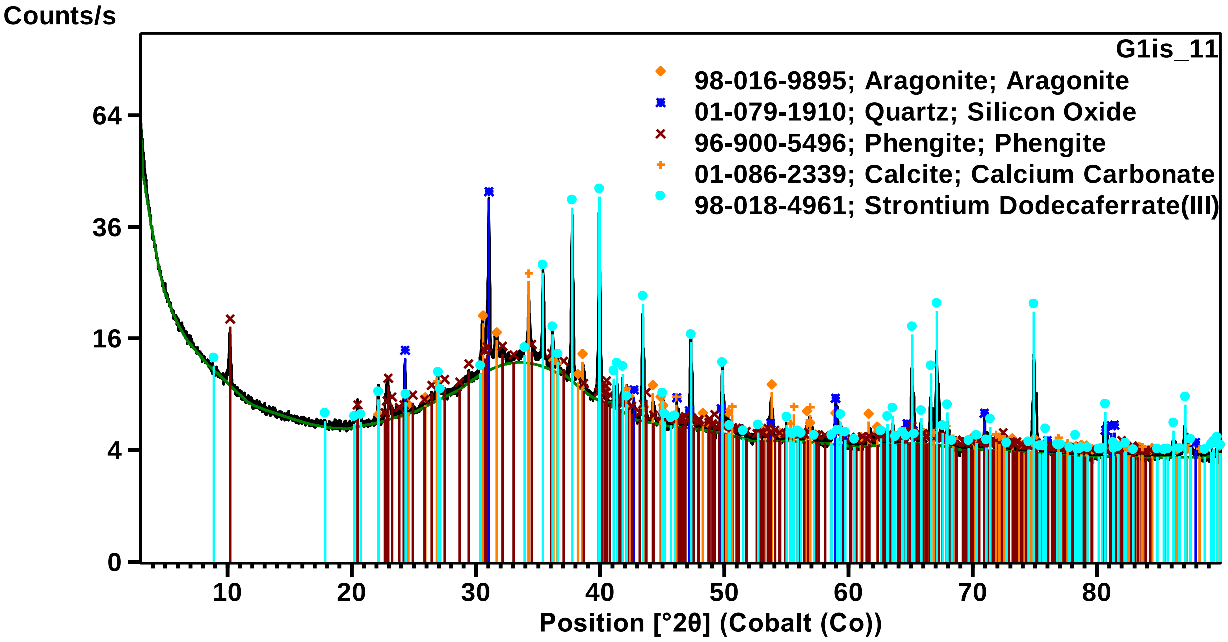

Figure S5. Full-scale XRD profile of the **G1is_11** composite.

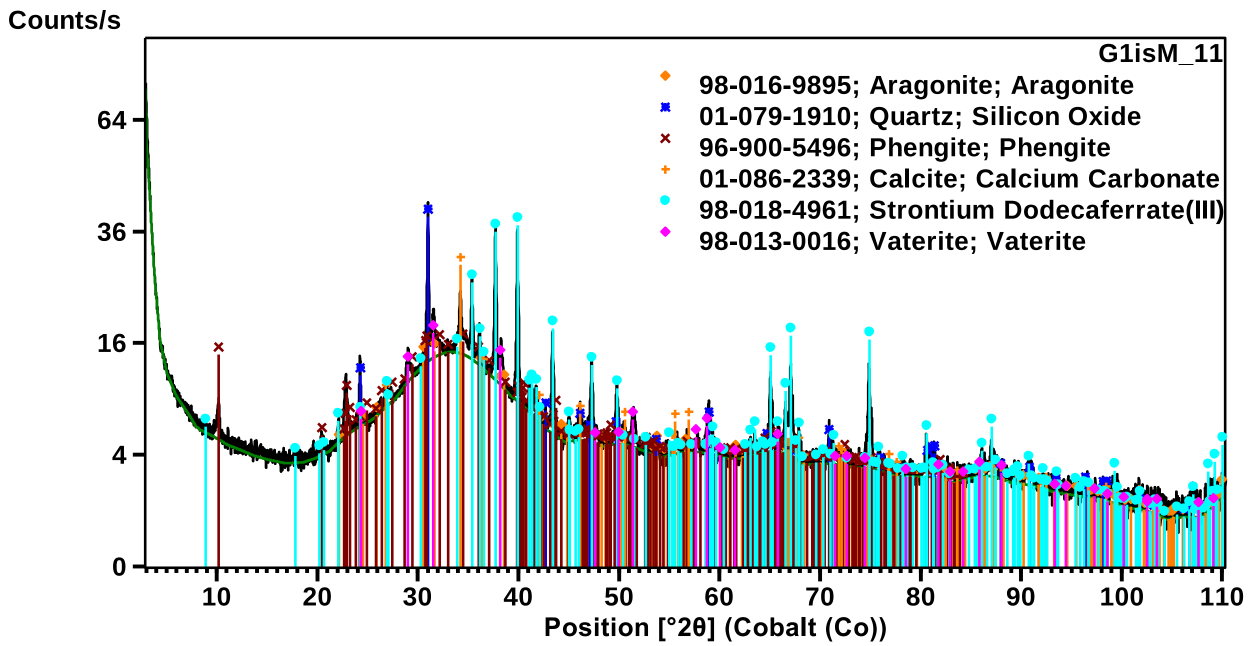

Figure S6. Full-scale XRD profile of the **G1isM_11** composite.

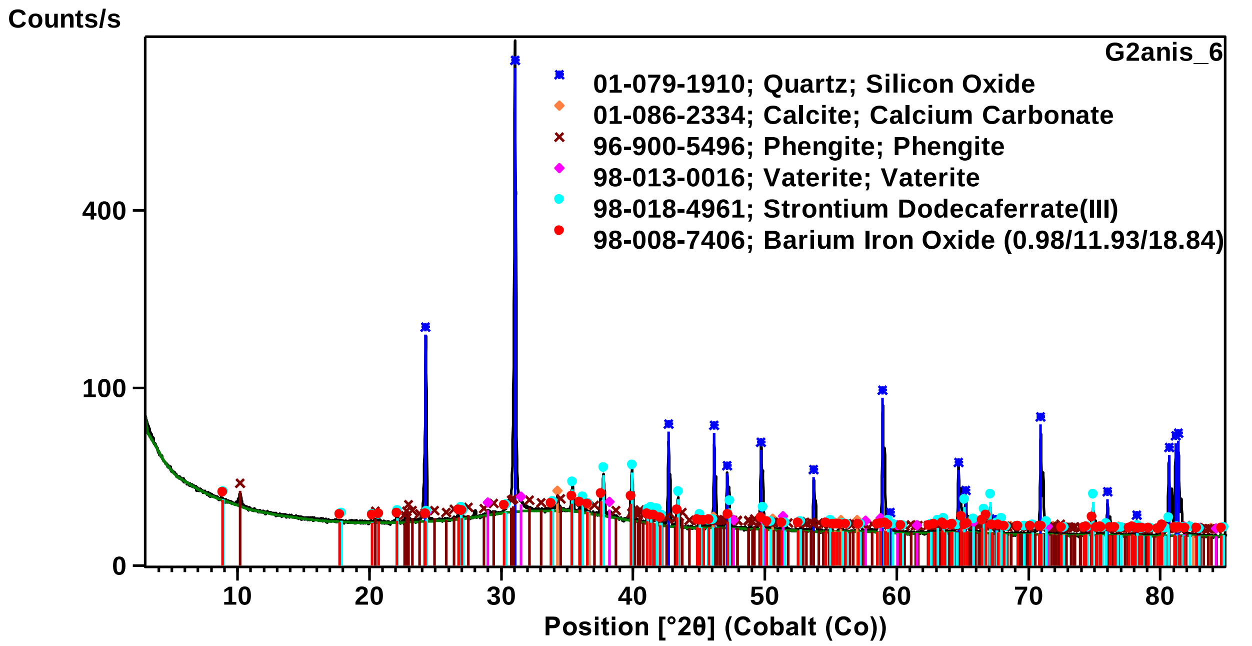

Figure S7. Full-scale XRD profile of the **G2anis_6** composite.

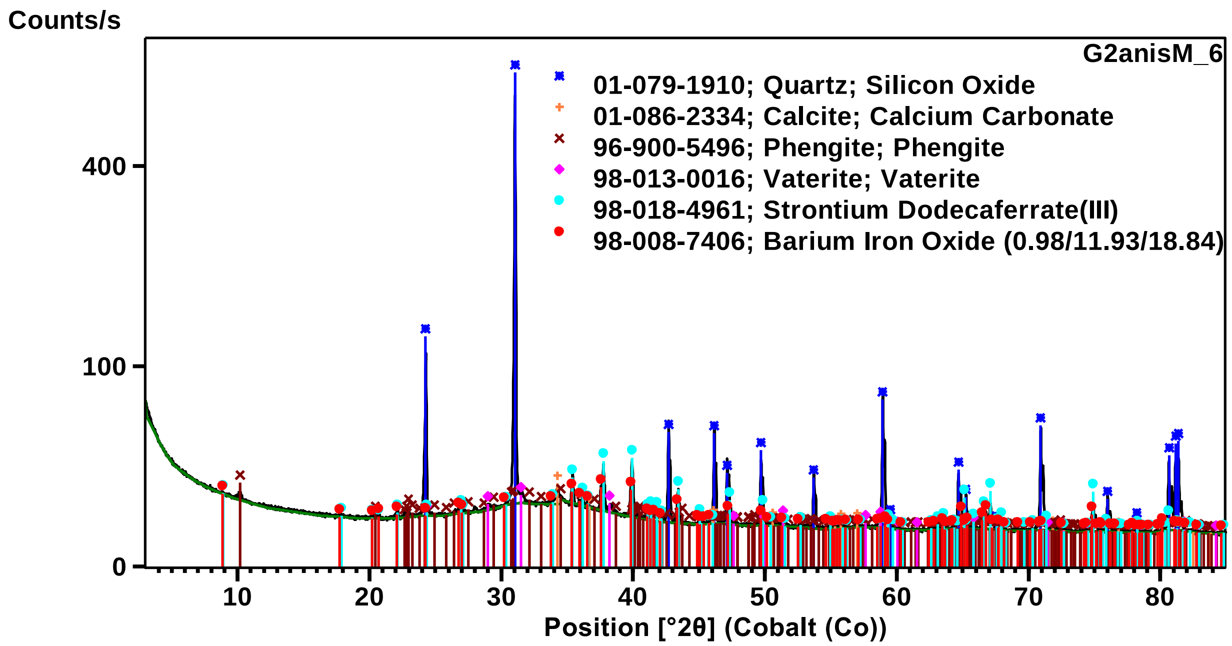

Figure S8. Full-scale XRD profile of the **G2anisM_6** composite.
